# Medical students’ perception of learning from patient encounters in primary health care; a qualitative interview study

**DOI:** 10.1186/s12909-023-04923-9

**Published:** 2023-12-08

**Authors:** Eva Öhman, Eva Toth Pal, Håkan Hult, Gunnar H Nilsson, Helena Salminen

**Affiliations:** 1 Division of Family Medicine and Primary Care, Department of Neurobiology, Care Sciences and Society, Karolinska Institutet, Alfred Nobels Allé 23, Huddinge, SE-141 83 Sweden; 2 Department of Clinical Science, Intervention and Technology, Karolinska Institutet, Stockholm, Sweden; 3 Academic Primary Healthcare Centre Stockholm, Stockholm, Sweden

**Keywords:** Primary health care, Medical education, Medical students, Clinical placement, Learning from patients

## Abstract

**Background:**

Clinical practice gives medical students opportunities to develop clinical skills and to gain insight into their future profession as a physician. Students in the medical programme at Karolinska Institutet in Sweden had clinical practice in primary health care in nine of their 11 semesters. The aim of this study was to explore medical students’ perceptions of learning from patient encounters in a primary health care context.

**Methods:**

The study was a qualitative inductive interview study. The 21 participating medical students were from their 3rd, 4th and 5th (final year) year of the study programme. A semi-structured interview guide was used. The data analysis was performed with qualitative content analysis.

**Results:**

The overarching theme of the study was: The individual patient encounters are the key to learning in primary health care. The patient encounters presented both useful opportunities and challenges that could contribute to the students’ professional development. The following four categories were found: 1. Patient encounters in are instructive, rewarding and challenging. Practising in primary health care provided experience in meeting and communicating with a wide variety of patients. Students described it being challenging to trust in their own clinical competence and feeling a responsibility towards the patients. 2. Encounters with patients in primary health care provide opportunities for gradual professional development. Students had the opportunity of increasing independence based on their level of clinical competence. They experienced a progression in their professional development after each period in primary health care. 3. A committed supervisor plays a significant role in learning. Committed supervisors who set aside time for supervision, offered support, and encouraged the student, played an important role in the student’s learning. 4. Learning in primary health care and learning in hospitals complement one another. It could be difficult for the students to sort out exactly where they learnt different things as they perceived that learning in primary health care and in hospitals complemented one another.

**Conclusions:**

The students’ encounters with authentic patients in primary health care gave them recurring opportunities to develop communication skills and to be trusted to work on their own under supervision, giving them guidance on their way to becoming future physicians.

**Supplementary Information:**

The online version contains supplementary material available at 10.1186/s12909-023-04923-9.

## Introduction

Clinical placements are the practical foundation of medical student development towards a future profession as a physician. Patients have a central role in students’ clinical learning. Medical students need to acquire clinical competence and a “state of mind that includes confidence, motivation and a sense of professional identity” [1]. The professional identity has been identified as when the student “comes to think, act and feel like a physician” [2]. Authentic patient encounters may be an important contribution to this identity formation. Primary healthcare (PHC) is a more and more emerging part of medical education. Students in clinical practice in PHC appreciate the challenge of being confronted with various patient problems and the clinically complex disease profiles the patients present, and the close contact with patients [3]. It has been demonstrated that students were generally positive about being able to develop their autonomy and decision-making skills under supervision in PHC [4]). Students appreciate being trusted to actively and autonomously practise dealing with patients to the extent commensurate with the level of their education [4, 5]. One previous study revealed the desire of students to feel welcome in the working group and to be part of the team [4]. Students who feel comfortable in their clinical learning environment also participate more actively in encounters with patients [6]. In the study of van der Zwet et al. the authors explored students’ learning in PHC from a sociocultural perspective. The findings of the study showed that for learning and developing a professional identity in a clinical learning environment, the students needed space for interaction with patients, supervisors and other professions [7]. This was found to have an important impact on the student’s experience of their student role.

Previous studies have focused mostly on the students’ relationship with supervisors and their perception of clinical learning environments. There are very few studies assessing students’ perception of learning from patient encounters in a clinical context. [8]

The aim of this study was to explore medical students’ perceptions of learning from patient encounters in a PHC context.

## Materials and methods

### Design 

The study was a qualitative interview study. A semi-structured interview guide was used. A qualitative inductive content analysis was performed on the material based on Graneheim and Lundman [9].

### Pedagogical theoretical framework

A community of practice (CoP) is a description of a community of people, members, with something in common, such as the same purpose and goals of the community. A CoP consists of a core of members with knowledge and skills that are in demand within the community. Around the community core there are beginners or learners, who are on their way to achieve the knowledge and skills required for the community and to become full members of the community [10, 11]. These learners are in a learning process and can be described as having legitimate peripheral participation. Legitimate peripheral participation means that a learner participates in a current ongoing activity in a community. A learning situation could be described as something that occurs in the relationship between a learner and other members of the same community at different stages of membership. During the learning process, the learner moves towards full participation in the community [11]. Experience Based Learning (ExBL) is related to CoP [12]. ExBL is a model of learning by experience where learning includes the whole person with her/his intellect, feelings, and senses [13]. To give an experience meaning, ExBL is based on three elements, “reflecting, evaluating and reconstructing” [13]. ExBL can be used to understand how the students’ clinical learning can take place in a learning environment that is also a workplace, such as a PHC centre. A learning situation at a PHC centre often includes a student, an authentic patient and a doctor who is there to support and challenge the student in a safe leaning environment [14]. ExBL is a model with the aim of helping students, by increasing challenges, to take a “step outside their comfort zone” [15]. If students are encouraged to step outside their comfort zone, the next step may be “the zone of proximal development” [16]. The zone of proximal development is “the distance between the actual developmental level as determined by independent problem solving and the level of potential development as determined through problem solving” [16]. The zone of proximal development was hypothesized in this study to be a component that may be of special importance in the clinical context of PHC.

### Context

In the study programme in medicine at Karolinska Institutet in Stockholm, Sweden, students have regular clinical placements in PHC as part of their education. Family medicine in Sweden is organised in PHC centres and are collectively referred to as PHC, responsible for first-line health care for the entire population and funded by public health insurance.

In Sweden an academic year contains two semesters, one in spring and one in autumn. The family medicine clinical placements run through the programme during 9 of its 11 semesters. Most of the students’ clinical placements take place on hospital wards. For the medical students who participated in the study, placement periods at PHC centres varied from four to seven days depending on the semester. Clinical placements in PHC are designed to provide the student with the opportunity to meet patients in authentic settings with the level of supervision based on their current level of education. During these patient encounters, medical students practice both clinical skills and communication skills based on the intended learning outcomes of the course. From the start of the programme, medical students practise early professional contact with patients based on a communication skills process model [17, 18]. In subsequent semesters, they gradually work on achieving a greater degree of autonomy in dealing with the entire patient encounter themselves with the support of a supervisor. Medical students from the third year on most often start the encounter with the patient themselves, after which they discuss their findings and the possible diagnoses and further investigations with their supervisor. The encounter is concluded with the supervisor and student joining the patient, to end the consultation and discuss further treatment in a dialogue with the patient.

### Population

Medical students in year three to fifth and final year of the programme were invited to participate in the study. Information about the study and the opportunity to participate was distributed via emails to students in the spring semester of 2020. Third-year students were invited to the study in conjunction with a seminar in family medicine. Any medical student who had an experience of at least one clinical placement in PHC was invited to participate. In total, 21 respondents were interviewed (Table 1).

### Data collection

Data collection took place by interviewing 21 medical students. The interviews were designed as semi-structured qualitative interviews with open ended questions in the interview guide (See Appendix 1). The interviews were conducted both as group interviews and individual interviews with the aim of obtaining as rich data material as possible.

The interviews were distributed as follows: Three group interviews and 11 individual interviews were performed in total. The three group interviews and two of the individual interviews were conducted in physical meetings in premises belonging to Department of Neurobiology, Care Sciences and Society, Division of Family medicine and Primary care. An observer was present during one of the group interviews. The nine individual interviews were conducted over the mobile phone as it was inappropriate to meet in person at the time due to the COVID-19 pandemic. The interviews lasted between 22 min and one hour.

All interviews were audio recorded and transcribed verbatim. The interviewer’s relationship with the study participants began at the first contact when the participants expressed interest in participation and ended in connection with the interview. Saturation was discussed and the material was considered saturated when no significant new data emerged in the interviews.

**Table 1:** Number of interviews and background data on the participating students

Interview method	Interviews (n)	Participants (n)	Semester	Women (n)	Men (n)
Group interview with 6 respondents	1	6	5	5	1
Group interviews with 2 respondents	2	4	5, 7	3	1
Individual interviews	11	11	7, 9, 11	8	3
In total	14	21		16	5

### Method of analysis

Qualitative content analysis with an inductive approach was used [9]. Meaning units were identified, condensed and coded. Three of the authors (ETP, HS, EÖ) individually coded all but three interviews. The last three interviews were coded by a single author, after which the coding was discussed with the two other authors. Individual codes were then jointly sorted into subcategories by the same three authors. Main categories were then formed. Finally, a theme was constructed that was prevalent throughout the material. The process of analysing interviews was performed manually without the use of a computer coding program. The COREQ checklist was followed and it is attached as a supplementary file.

### Ethics

The study was assessed and received an advisory opinion by the Regional Ethical Review Board in Stockholm, Sweden. (Ref. no. 2011/382 − 31/4. Ref. no. 2012/289 − 32)

Each student gave their informed consent to participate in the study. Students interviewed in person signed a consent form at the interview. Students interviewed over the mobile phone emailed their informed consent before the interviews took place.

## Results

A total of 21 students participated in the study, five men and sixteen women. Distribution of the participants is shown in Table 1.

### Overall theme: the individual patient encounters are the key to learning in PHC

The overall theme demonstrates the central role PHC patients had in the students’ development and training in clinical skills. An authentic clinical environment and meeting authentic patients with common medical conditions and symptoms having not yet been diagnosed combined with support from supervisors made the students feel that they could take one more step in their professional development each time they returned to their clinical placements in PHC. Clinical skills such as communication skills and interpersonal skills for dealing with patients were practised. The students received training in administration and medical record keeping. They perceived taking individual responsibility for the patients that they handled although everything they did was double checked by their supervisor.

Table 2 shows an overview of the theme, main categories and subcategories of the study.

**Table 2:** Theme Main categories and subcategories

Theme: The individual patient encounters are the key to learning in PHC	
**Main categories**:	**Subcategories**:
Patient encounters in PHC are instructive, rewarding and challenging	Provides training in interacting with patients
	Patient interactions in PHC can be emotionally challenging
	A more holistic view of the patient appears in PHC
Encounters with patients in PHC provide opportunities for gradual professional development.	One learns to take on a professional role
	The medical student’s educational progression is made visible in PHC
	The medical student’s experience of broad learning in PHC
A committed supervisor plays a significant role in learning	Committed supervisors enable the medical student’s development
	Committed supervisors are crucial to learning during observation
Learning in PHC and learning in hospitals complement one another	Switching between PHC and hospitals is instructive
	Structural prerequisites for learning in PHC and in hospitals
	Primary care provides breadth; hospital care provides specific depth

### Patient encounters in PHC are instructive, rewarding and challenging

The students explained that it was in encounters with patients in PHC that they gained experience and familiarity with meeting and communicating with different types of patients. Students found the communication skills process model (with patient’s, doctor’s and a shared agenda) to be helpful, as this provided a good structure for the encounters. This model allowed the patient to expand or clarify if anything had been missed or misunderstood. The ambition was expressed that, even if it was sometimes unavoidable to steer the conversation in a given direction, the patient should still be able to explain their agenda and feel that they were being listened to. Several interviewees described an awareness that the stated reason for an appointment might be concealing something else.

The patient’s story was the focus in the meeting, answers to laboratory tests often came later.

“*And of course there’s this sort of detective work that often means that you have to learn to listen for things to understand a problem that might not be obvious straight away.” (Individual interview. Stud V. Semester 11, F)*.

Some questions in taking the patient’s medical history might concern personal or intimate matters. Students felt it was important to ask these questions, but some students expressed an insecurity about asking certain questions.

*“It’s better, less of a risk, to offend someone by asking than to miss something serious by not asking.” (Individual interview. Stud C. Semester 9, M)*.

Students described different ways of relating to the patients. If a patient was perceived to be anxious, the student could show empathy and adapt their responses to the patient’s anxiety. If an appointment did not turn out as the patient expected, students had their own strategies for dealing with the situation.

Students felt that, in PHC, the approach to the patient was more holistic than in the hospital context; encounters were more personal, and it was easier to discern the person behind the patient. Students considered it to be positive when patients shared information about social factors during an appointment. This made students consider the patient’s home environment and how health care measures might work in the patient’s home.

*“…that you learn so much about people just because they arrive from everyday life dressed in their regular clothes and, well, they discuss their lives more than they would in a hospital where they are wearing hospital gowns, you know, so it becomes more personal.” (Group interview. Semester 5, F)*.

Students explained that meeting patients alone was not always entirely positive. Some doubted whether their competence was good enough, they were concerned and nervous about not being able to establish confidence, calm and a sense of trust. Some students mentioned that they were worried about missing something in a situation where their actions had a bearing on someone else’s health. They were interested in satisfying the patient, even if the problem could not be solved then and there. They felt uneasiness when they were unable to help the patient.

*“You began to feel like, wow, this is for real. I’m actually going to do something that plays a major role for somebody else here.” (Individual interview. Stud Y. Semester 9, F)*.

*“If it was hard afterwards, I could think… look at it like this…*

*there’s not a lot I can do to reduce her pain” (Individual interview. Stud U. Semester 7, F)*.

### Encounters with patients in PHC provide opportunities for gradual professional development

According to the students, their own development progressed with each placement in PHC. The regularly recurring periods in PHC facilitated learning and development for the students. The broad patient base in PHC, with the most common symptoms and conditions, was perceived as highly educational in terms of dealing with patients clinically and organisationally. The wide range and randomness of causes for visits to PHC made them prepared to deal with the unexpected. The students discovered that more serious cases could be hidden among the common non-life-threatening complaints. It was important to be able to know when a patient should be sent to hospital.

*“In any case, I feel that I’ve begun to get a sense of when I should send a case to radiology and when not.” (Individual interview. Stud Y. Semester 9,F)*.

Students underlined that having the confidence of supervisors and patients to deal with a patient visit more and more independently was highly developmental. A private examination room and collecting the patient themselves, discussing and proposing a strategy going forward, all of this was perceived by students as enabling them to try out the role of doctor. In encounters with patients, they felt they had a responsibility not to miss anything important. PHC was perceived as somewhere they could get good training before graduating.

*“…I often find that, after a placement at a health centre, it feels like I’ve grown a little more into my future role as a doctor.” (Individual Interview. Stud IPA. Semester 7, F)*.

### A committed supervisor plays a significant role in learning

Medical students described supervisors in PHC most often to be more senior and more committed to supervision than the supervisors they had met in hospitals. They felt that supervisors in PHC offered good support and sometimes pushed them a little more. Interviewees spoke about the importance of supervisors setting aside adequate time, having commitment and an interest in supervision, and having a pedagogical approach. While medical students generally preferred to have the same supervisor throughout their placement, some stated that a change of supervisor and a change of the PHC centre for their placement could reveal contrasts and different approaches, for better or worse.

*“…this encouraged you to take responsibility and shape up in your learning and so on, and it’s very instructive, you know, as you can really test yourself.” (Individual interview. Stud C. Semester 9, M)*.

The medical students said that observation could also be instructive when one’s own skills were insufficient in the situation in question. Early in the programme, observation offered an insight into the process of a doctor’s appointment and communication between doctor and patient. The students were able to see how doctors performed different examinations and at the same time were given practical examples of how to use different instruments. Committed supervisors gave reasons for their actions and, depending on the supervisor, observation could be an interesting learning situation.

During observation with different doctors, students could compare different ways of dealing with patients. This was viewed as developmental and offered students opportunities to cherry-pick the elements they felt worked well.

*“…so you also see that everyone does things differently and you can kind of put together the things you think have worked well.” (Individual interview. Stud W. Semester 9,F)*.

Some students said that they felt more involved when they met the patient together with their supervisor. However, they also said that observation dictated passivity and it was not rewarding if the patient’s condition could not be linked to the intended learning outcomes of their current course. The majority of the students were of the opinion that seeing patients themselves was almost always better than observing.

### Learning in PHC and learning in hospitals complement one another

Students felt that learning in PHC and learning in hospitals complemented one another and that all clinical placements were relevant and meaningful. It was difficult for them to pinpoint where different kinds of knowledge came from; students described it as developmental to switch between PHC and the hospital. There was a view that experience they gained from meeting patients in PHC made their meetings with patients in the hospital easier.

*“Then in terms of [patients’]thoughts, worries and wishes, we’ve had the chance to practice a great deal and this is, of course, something that I also bring to the interaction with patients in hospital” (Individual interview. Stud B. Semester 9,F)*.

In hospital wards, patients had more well-defined problems. There was a more limited disease profile that was easier to focus on. Patients in PHC offered greater breadth and common diseases. These were often simpler cases and the vital thing was to rule out any medical condition that needed to be dealt with quickly. Interviewees said that, in hospitals, there was more time to get information about patient histories, to read medical records and check test results. Many students felt that in PHC there was greater time pressure and less time to prepare for appointments, but there were also students who did not feel any time pressure in PHC at all.

*“…well, I find primary health care interesting because (…) you have a completely different level of preparation than in inpatient care and, in my experience, less opportunity to prepare.” (Individual interview. Stud A. Semester 7, M)*.

## Discussion

The individual patient encounters were the key to medical student learning in PHC according to the results from this interview study with medical students. Authentic patient encounters in an authentic clinical environment under safe supervision provided experience and valuable practice in clinical work. When the supervisor felt trust in the student, the encounter could be partially handled by the student. The students said that a progression towards their future profession took place after each period of their clinical practice in PHC.

The responses from the students indicated that encounters with patients in PHC were instructive, rewarding and challenging. Students described that in PHC they could meet and learn about a patient as the complex person she/he is in that person’s own social context. Through interaction in each patient encounter, the students in our study learned to lead the conversation, to minimise the risk of misunderstanding, but also to avoid overlooking symptoms that might need prompt action. These findings in our study together with findings in previous studies, carried out both in PHC [18, 19] and hospitals [6, 20], show how crucial authentic clinical learning situations are for the student’s opportunity to develop new competence and knowledge. In our study, PHC could provide a learning environment that offered students opportunities to meet a diversity of authentic patients in an authentic clinical setting. PHC gave students space to practice and develop clinical skills in patient encounters on their own but still under supervision.

The student’s individual encounters with patients can be both challenging and rewarding. Our findings show that students could sometimes feel insecure when they handled parts of the encounter on their own. They felt concerned about missing something important or that their competence would not be sufficient. This is both a challenging and rewarding situation, where the students sometimes found themselves slightly outside their comfort zone. They felt responsible for their patients which must be the best driving force for learning. It can be what Vygotsky described as the zone of proximal development, a space in which the individual is challenged to develop further [21, 16]. This zone of proximal development has also been described as the space between what learners can do alone, and what they can do with help from others, in this case with the help of their supervisors [22]. If the challenge was handled well by both the student and supervisor, it could be a positive experience and a new level of clinical competence could be achieved. The feelings of growing with the challenges can be experienced as rewarding. By using a learning process such as the ExBL model, the new experience can be given meaning by the student’s reflection, evaluation and restructuring. The new knowledge is then incorporated with already existing knowledge [12]. The learning process is repeated each time the student returns to PHC and meets new patients. The new encounters with the patients will be based on their new level of competence. PHC seems to be a good place for vertical concrete integration throughout the medical programme that may be used to visualise the student’s progress.

Findings in this study show that encounters with patients in PHC provide opportunities for gradual professional development. Being an active student in a clinical environment in PHC helps the student to prepare to meet patients with different problems and agendas. It prepares for the unexpected, and students realise that the ordinary can conceal the unusual.

With increasing clinical competence, the student moves closer into the professional community that constitutes the medical profession [11].

The gradual training from acting on their own during clinical learning contributes to the students’ progression [18]. The students in our study themselves noticed a progression after each period in PHC. Our study also shows that the students felt a sense of responsibility towards the patient. A connection between the student acting on their own and a sense of responsibility towards the patient has also been shown in a previous study [23]. A connection that showed that the opportunity of acting on their own is one of several components in the student’s progression towards the future profession. During the interviews in our study, the students often said that they could work independently, which can be seen as an expression of the supervisor and patient showing the student trust so that the student could carry out parts of the patient encounter on their own. Trust from supervisors to interact alone and having their own examination room are important för the student’s construction of their future professional role [23, 5]. Having access to a room of their own with possibilities to interact alone with the patients shows trust from supervisors and confirmation of the community in one’s journey toward becoming a professional doctor [11]. According to our findings, a committed supervisor constitutes an important part of the student’s professional development and plays a significant role in learning. The student must not only develop their clinical competence but also create a professional identity. Students’ need for support in creating their professional identity has also been addressed in previous studies [24, 25]). The findings in our study show that a good supervisor gave space and encouragement, and pushed the students to act themselves, to take responsibility, and to step out of their comfort zone. Feedback from supervisor to student is an important component in the student’s progression and a previous study showed that students wanted feedback from the supervisor adapted to their level of education [5]).

The feedback from the supervisor could also play an important role in the student’s learning process by helping the student reflect on and evaluate their experience both during the ongoing period and when the period was over. Clinical learning in PHC and in hospitals compl**e**ment each other according to findings in our study. Learning from encounters with patients and use of structured communication from PHC was helpful when applied to meetings with patients at the hospital. Patients in hospitals often have known conditions, while patient encounters in PHC often involve more uncertainty and the ruling out of the possible dangerous causes behind the symptoms. The students’ thoughts about where they acquired knowledge about different things and skills, whether from the hospital wards or from PHC were difficult to identify in our study. This could show that learning in hospitals and learning in PHC are important complements to each other. There was only one exception: Communication skills with patients. Students learned these in PHC and described having good use of them in hospitals.

### Strengths and limitations

The fact that students had been on a placement in PHC just before the interviews, and therefore had recent experiences to draw on, was clearly a strength of the study and increases its credibility. To further increase the credibility of the study, data was coded and categorised in order to ensure that the material which concerned the research question was covered as well as possible. All codes, categories and themes from all interviews were reviewed by three of the authors (EÖ, ETP, HS) and discussed until a consensus was reached.

Three of the authors had previous experience of supervising medical students during periods in PHC, which might provide a level of understanding that could affect how interviews were interpreted. It could also provide a strength to those interpretations.

The gender imbalance, with women overrepresented among participants, does not correspond to the actual gender balance in the programme. The fact that many of the interviews were conducted over the mobile phone due to the COVID-19 pandemic may also be a weakness. Interviews via mobile phone could sometimes be somewhat disturbed by background noise.

## Conclusion

Regularly recurring practice periods with authentic patient encounters in PHC were viewed by the students as the key to learning communication and clinical skills and gaining experience of handling a broad range of patients. Experience of feeling responsible for handling the patient encounter and sometimes feeling uneasy as to whether the level of competence was sufficient at the time was perceived as challenging. Committed supervisors were a source of security, giving students the confidence to act on their own. The students themselves were able to notice a progression in their development towards their future profession after each occasion in PHC.

### Electronic supplementary material

Below is the link to the electronic supplementary material.

**Supplementary Material 1:** COREQ checklist for the study

## Data Availability

The datasets generated and/or analysed during the current study are not publicly available due to ethical and legal restrictions since it contains potentially identifying and sensitive information that compromises the participants ‘confidentiality and violates the data protection policy of Karolinska Institutet. Requests should be made to the first author, Eva Öhman, eva.ohman@ki.se. Transcribed interviews (in Swedish) are stored at Karolinska Institutet, Department of Neurobiology, Care Sciences and Society. The data set is not publicly available The data that support the findings of this study are available on request (in English translations) from registrator@ki.se pending approval of the Regional Ethical Review Board.

## List of abbreviations

CoP: Communities of practice
ExBL: Experience based learning
PHC: Primary healthcare
