# Supplementary material for: Medical students’ perception of learning from patient encounters in primary health care; a qualitative interview study
Source: BMC Med Educ. 2023 Dec 8;23:935. doi: 10.1186/s12909-023-04923-9 (PMC10709937; doi:10.1186/s12909-023-04923-9)
Supplement: Supplementary file 1 — Supplementary Material 1: COREQ checklist for the study [file 12909_2023_4923_MOESM1_ESM.docx]

**Appendix 1.**

**Interview guide to student interviews**

**Title of Project: Medical students’ Perception of Learning from Patient Encounters in Primary Health Care; A Qualitative Interview Study**

The purpose of this project is to explore medical students´ perceptions of learning from patient encounters in a primary health care context. The focus of this interview is:

**What do I learn as a student from patients in primary health care?**

In this interview, we will talk about your experiences and thoughts based on your interactions with patients in primary health care.

* What have the interactions with the patients in primary care taught you?

- What lessons have you learned from working at the health centre?
- Do you see any differences in what interactions with patients give you at the health centre compared with the hospital?

* What specifically did you learn

* when you had your own patient consultations?

* when you observed during “sit-in observations”?

* Were you given the opportunity to handle patients with complex clinical presentations? Can you describe this?

* Were there any questions you found difficult to ask? (or* Was there anything you thought was difficult to bring up with patients?

* Do you feel that you could glean experience from primary care that is useful in interactions with hospital patients?

* Did you change anything in your way of interacting with patients in hospital based on experiences from primary care? Can you describe this?

* How would you summarise all of your primary care periods from a learning perspective?

* Did you observe anything special when you saw patients out in the waiting room or met them in the hallway?
